# Chemical Genetics of Acetyl-CoA Carboxylases

**DOI:** 10.3390/molecules18021704

**Published:** 2013-01-28

**Authors:** Xuyu Zu, Jing Zhong, Dixian Luo, Jingjing Tan, Qinghai Zhang, Ying Wu, Jianghua Liu, Renxian Cao, Gebo Wen, Deliang Cao

**Affiliations:** 1 Institute of Clinical Medicine, the First Affiliated Hospital, University of South China, Hengyang 421001, Hunan, China; 2 Institute of Translational Medicine & Department of Laboratory Medicine, the First People’s Hospital of Chenzhou, 102 Luojiajing Road, Chenzhou 423000, Hunan, China; 3 Department of Microbiology, Immunology and Cell Biology, Simmons Cancer Institute, Southern Illinois University School of Medicine, 913 N. Rutledge Street, Springfield, IL 62794, USA

**Keywords:** acetyl-CoA carboxylase, fatty acid biosynthesis, ACC chemical genetics, ACC inhibitors, cancer therapy

## Abstract

Chemical genetic studies on acetyl-CoA carboxylases (ACCs), rate-limiting enzymes in long chain fatty acid biosynthesis, have greatly advanced the understanding of their biochemistry and molecular biology and promoted the use of ACCs as targets for herbicides in agriculture and for development of drugs for diabetes, obesity and cancers. In mammals, ACCs have both biotin carboxylase (BC) and carboxyltransferase (CT) activity, catalyzing carboxylation of acetyl-CoA to malonyl-CoA. Several classes of small chemicals modulate ACC activity, including cellular metabolites, natural compounds, and chemically synthesized products. This article reviews chemical genetic studies of ACCs and the use of ACCs for targeted therapy of cancers.

## 1. Introduction

**Figure 1:**
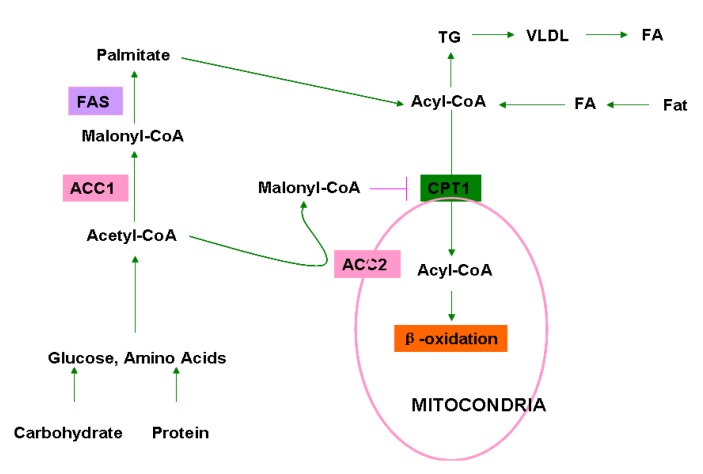
Roles of ACC1 and ACC2 in cellular fatty acid/lipid metabolism. Dietary fats, carbohydrates, and proteins are primary sources of fatty acids (FA), glucose, and amino acids. FA is converted to acyl-CoA in liver, and glucose and amino acid are converted to acetyl-CoA in the liver and adipose. In the cytosol, acetyl-CoA is carboxylated to malonyl-CoA by ACC1 which is utilized by fatty acid synthase (FAS) to produce palmitate, the end product of fatty acid biosynthesis. Palmitate is further converted to various acyl-CoA by elongase and desaturase. Acyl-CoA is shuttled into the mitochondria through carnitine/palmitoyl-transferase 1 (CPT1) for β-oxidation. Acyl-CoA could also be utilized for the synthesis of triglycerides (TG) in adipose tissues. Acetyl-CoA is also carboxylated by ACC2 at the mitochondrial membrane to generate malonyl-CoA, which inhibits acyl-CoA transfer and oxidation in mitochondria.

Acetyl-CoA carboxylases (ACCs) are biotin-dependent enzymes in fatty acid *de novo* biosynthesis that irreversibly catalyze carboxylation of acetyl-CoA to malonyl-CoA [1,2,3,4]. This reaction proceeds via two continuous steps: ATP-dependent carboxylation of biotin with bicarbonate as a donor of CO_2_ and carboxyl group transfer from biotin to acetyl-CoA to form malonyl-CoA. The product malonyl-CoA is dual-functional depending upon its subcellular location. In cytosol, malonyl-CoA is a building block of long chain fatty acids, but in mitochondria, malonyl-CoA serves as an inhibitor of fatty acid β-oxidation by inhibiting the transfer of fatty acyl from acyl CoA to carnitine by carnitine acyltransferase, also known as carnitine palmitoyltransferase-I (CPT1). In humans and mammals, there are two isoforms of ACCs: ACC1 (ACC-α) with 265 kDa and ACC2 (ACC-β) with 280 kDa. ACC1 is localized in the cytosol and its product malonyl-CoA is utilized for fatty acid biosynthesis. In contrast, ACC2 is located mainly in mitochondria and the produced malonyl-CoA serves as an inhibitor of fatty acid β-oxidation. Therefore, ACC1 is enriched in lipogenic tissues, such as the liver, adipose and lactating mammary gland, where it catalyzes the biosynthesis of long-chain fatty acids. On the contrary, ACC2 is highly expressed in oxidative tissues, such as the skeletal muscle and heart, regulating fatty acid β-oxidation [5]. However, ACC1 and ACC2 are both highly expressed in the liver where fatty acid synthesis and oxidation are both active [6]. Therefore, ACCs play a key role in the synthesis and metabolism of fatty acids (Figure 1).

ACCs are highly conserved in their amino acid sequence and function in most living organisms, including archaea, bacteria, yeast, plants, rodents, and humans. In mammals and most eukaryotic organisms, ACCs are a multiple domain polypeptide consisting of three domains: biotin carboxylase (BC), biotin carboxyl carrier (BCCP), and carboxyltransferase (CT), but in prokaryotic cells, these domains exist as separate units. The BC domain/subunit catalyzes carboxylation of the N1 atom in the ureido ring of biotin covalently linked to a lysine residue in BCCP with bicarbonate as a donor of carboxyl group, and the CT domain/subunit catalyzes the transfer of the carboxyl group from the N1 atom to the methyl group of acetyl-CoA [7]. The BC subunit itself exists as a dimer in solution. Crystal structure studies show that the yeast BC domain contains 20 β-strands (β1–β20) and 21 α-helices (αA–αU) and forms three sub-domains (A, B, and C) and an ATP-grasp fold [8,9,10,11,12]. The active site of the enzyme is located at the interface of the β-domain and cylinder [11], and the β-domain keeps an open conformation for substrate binding or product release, but is closed during the catalytic process. The dimer of CT domain in yeast is the only structure currently available, in which two monomers of the CT domain form a CT domain dimer by a side-to-side reverse arrangement. A yeast CT domain monomer consists of 24 α-helices and 29 β-strands, forming two sub-domains (N- and C-domains) intimately associated with each other. The catalytic pocket is formed by small β-sheets and α6 helix of β-β-α superhelix in the two domains and the active site is located at the middle site of the interface of N- and C-domains in the dimer. Conserved residues in the active site, including Arg 1954 and Arg 1731 are important for carboxyl group recognition of malonyl-CoA [13]. The *E. coli* BCCP subunit contains the essential biotin that binds covalently to lys 35 in the C-terminus, and the integral BCCP tends strongly to aggregate [14,15]. The interaction of BCCP with BC is determined by N-terminus (residues 1-30) of BCCP, and the complex of BC and BCCP could be biotinylated *in vitro*. Readers are referred to a recent review article [16] for more details.

## 2. Acetyl-CoA Carboxylase Genes, Expression Regulation and Posttranslational Modifications

In mammals, ACC1 and ACC2 are encoded by two different genes. Prokaryotes and plants have multiple ACC subunits that are composed of several polypeptides encoded by distinct genes. For instance, ACCs in *Streptomyces coelicolor* (*S. coelicolor*) are comprised of α-subunit containing BC and BCCP domains and β-subunit (CT domain) encoded by *accA1*(*A2*) and *pccB* genes, respectively [17,18,19], but ACCs in archaeal *Acidianus brierleyi* are comprised of three subunits encoded by *accC*, *accB*, and *pccB* gene, respectively [20].

In mammals, ACC expression and activity is regulated at transcriptional, posttranslational, and metabolite-allosteric levels. At the transcriptional level, ACC1/2 expression is regulated by a group of transcription factors: sterol-regulatory-element binding protein 1 (SREBP-1), liver X receptor, retinoid X receptor, peroxisome-proliferation-activated receptors (PPARs), forkhead box O (FOXO), PPARγ, MyoD, Myf4 and Myf6 and PPARγ co-activator (PGC) and carbohydrateresponse element binding protein (ChREBP) [21,22,23,24,25]. A series of signaling molecules, such as glucose, insulin, and thyroid hormones affect the expression of ACCs through these transcriptional factors. For instance, high-carbohydrate diet induces ACC expression through ChREBP [26] or an insulin-mediated SREBP1 mechanism [27]. The ACC1 expression is also modulated at the translational level. In breast cancer cells, human epithelial growth factor receptor-2 (HER2) enhances ACC1 protein level through a PI3K/AKT/mTOR signaling-mediated translational mechanism [28].

Phosphorylation on several critical Ser residues in ACCs is a main posttranslational modification mode, affecting ACC enzyme activity (4). AMP-activated kinase (AMPK), activated by a variety of stress signals and adipokines, is a key kinase for the phosphorylation of Ser residues of ACCs under physiological conditions, which significantly inhibits the enzyme activities of ACCs through reducing Vmax and desensitizing citrate activation [29]. Phosphorylation of Ser residues in ACCs also potentiates the inhibitory effect of palmitoyl-CoA [30]. The sites of phosphorylation by AMPK include Ser79, Ser1200, and Ser1215 in ACC1 and Ser218 in ACC2. Ser79 in ACC1 and equivalent Ser218 in ACC2 are resides just prior to the BC domain, and their phosphorylation is sufficient to inhibit the activity of ACCs [29,31]. Protein kinase A (PKA) also phosphorylates Ser77 and Ser1200 in ACC1 [32,33], but the physiological significance remains to be determined. In addition, breast cancer protein 1 (BRCA1) interacts with ACC1 and prevents it from dephosphorylation at Ser 79 and Ser 1263 [34,35], suggesting a role of BRCA1 in the regulation of ACC1 enzyme activity. The ACC1 Ser 1263 is essential for the interaction of BRCA1 and ACC1.

## 3. Chemical Genetics of Acetyl-CoA Carboxylases

In addition to the conventional genetic and epigenetic regulation, ACC activity is also modulated by intracellular metabolites and proteins. In view of their central role in fatty acid biosynthesis and oxidation (Figure 1), ACCs have long been used as targets for the discovery of novel agents for the treatment of obesity, diabetes, and cancers [36,37,38,39,40,41,42,43]. A variety of small molecules that affect the conformational structure and enzyme activity of ACCs have been developed and extensively explored in preclinical and clinical trials in the past decades [7,44]. According to their origins, these ACC inhibitors are classified into natural and chemically synthesized small chemicals.

### 3.1. Cellular Metabolites and Proteins Modulating ACC Activity

Several cellular metabolites can induce ACC conformational alterations and modulate enzyme activity. Citrate, a precursor of acetyl-CoA, inhibits the binding of palmitoyl-CoA to ACCs and thereby stimulates the conversion of acetyl-CoA to malonyl-CoA [45,46]. Some anions, such as gisocitrate, malonate, sulfate and phosphate, promote polymerization and activity of ACCs. In contrast, palmitoyl-CoA, an end-product of fatty acid biosynthesis, props up the inactive conformation of ACCs and inhibits malonyl-CoA production [47]. Other long-chain fatty acids and acyl-CoA esters also show inhibitory effect on microorganism and rodent ACCs [48,49], and saturated acyl-CoA with 16~20 carbons possesses most inhibitory activity [50]. For instance, w-hydroxy-alkanedicarboxylic-CoA ester can reduce serum free fatty acid and triglyceride levels and body weight in experimental animals [51].

ACC activity is also modulated by some cellular proteins. Aldo-keto reductase 1B10 (AKR1B10), a NADPH-dependent xenobiotic reductase [52,53,54], binds to ACC1 and blocks its ubiquitin-dependent degradation, enhancing fatty acid synthesis [55,56,57]. Breast cancer protein 1 (BRCA1), a tumor suppressor, prevents ACC1 from dephosphorylation at Ser 79, inhibiting its enzyme activity [34,45].

### 3.2. Natural Acetyl-CoA Carboxylase Inhibitors

#### 3.2.1. Soraphen A

Soraphen A is a natural polyketide isolated from the myxobacterium *Sorangium cellulosum* [58,59]. Soraphen A contains an unsaturated 18-membered lactone ring, an extracyclic phenyl ring, two hydroxyl groups, three methyl groups, and three methoxy groups [60,61,62]. Soraphen A exerts inhibitory activity to ACCs by interacting with the BC domain of eukaryotic ACCs at an allosteric site and thus disrupting the oligomerization [63]. Soraphen A has been tested extensively as a broad-spectrum fungicide for agricultural applications [59] and used as a dominant selection marker for fungal transformation [64], but has no effect on the bacterial BC subunit [37,63,65].

#### 3.2.2. Andrimid

Andrimid is a hybrid non-ribosomal peptide-polyketide antibiotic that can block the carboxyl-transfer reaction. Andrimid has high selectivity for prokaryotic ACC and inhibits the biosynthesis of fatty acids with an IC_50_ at the sub-micromolar level. Andrimid has a hybrid non-ribosomal peptide polyketide scaffold that is acylated at the N-terminus and modified by a pyrrolidine dione moiety at the C-terminus. This class of molecules is widely distributed in nature and has received considerable attention after their cellular targets are discovered as ACCs [66]. Recently, andrimid was identified as a potent inhibitor of the CT step in the ACC-catalyzed carboxylation in bacteria.

The andrimid gene cluster from *Pantoea agglomerans* encodes an admT with homology to the arboxyltransferase (CT) β-subunit encoded by accD. *E. coli* cells with admT overexpression are resistant to andrimid. When AdmT and CT α-subunit are overexpressed in *E. coli* cells, an active heterologous tetrameric CT A2T2 complex is formed. andrimid-inhibition assays showed an IC_50_ of 500 nM for the A2T2 complex, compared to 12 nM for E. coli CT A2D2. These data suggest that AdmT confers resistance to andrimid [67] by functioning as an AccD homolog.

### 3.3. Chemically Synthesized Acetyl-CoA Carboxylase Inhibitors

#### 3.3.1. 5-(Tetradecyloxy)-2-furancarboxylic acid (TOFA)

As a representative of fatty acyl-CoA mimetics, TOFA reduces fatty acid synthesis by inhibiting ACCs after being converted to a CoA derivative (TOFyl-CoA) [1]. In cultured hepatic cells, TOFA inhibits lipid synthesis and TG secretion, and in animals TOFA lowers down plasma TG levels and body weight [68,69,70,71,72,73]. As a hypolipidemic agent, TOFA shows stronger inhibitory ability to ACCs than oleate, a natural fatty acid [74]. Interestingly, inhibition of fatty acid synthesis by TOFA leads to reduction of glycolysis by a secondary effect of a metabolite inhibitor of phosphofructokinase [75]. Our work has shown that TOFA induces apoptosis in lung cancer cells NCI-H460 and colon carcinoma cells HCT-8 and HCT-15 by inhibiting fatty acid/lipid synthesis, indicating its potential as an antitumor agent [76].

#### 3.3.2. CP-640186

CP-640186, an N-substituted bipiperidylcarboxamide, is a more potent, metabolically stable analog of CP-610431 [77]. CP-640186 is isozyme-nonselective and shows inhibitory activity to both ACC1 and ACC2 with an IC_50_ at 50 nM, reducing malonyl-CoA in lipogenic and oxidative tissues. Therefore, CP-640186 inhibits fatty acid synthesis and increases fatty acid oxidation, reducing body weight and improving insulin sensitivity [2]. In experimental animals, CP-640186 reduces triglycerides in the liver, muscle and adipose, and induces hyperinsulinemia triggered by high sucrose diet [78].

#### 3.3.3. ESP-55016

ESP-55016 is a ω-hydroxy-alkanedicarboxylic acid. After being converted into ESP-55016-CoA *in vivo*, ESP-55016 inhibits ACC activity. ESP55016 can inhibit both fatty acid and sterol synthesis *in vivo* and in primary rat hepatocyte culture [51]. ESP55016 increases serum HDL-C and β-hydroxybutyrate and reduce serum non-HDL-cholesterol (non-HDL-C), triglyceride, and non-esterified fatty acid levels in obese female Zucker rats [51]. ESP55106 also enhances [14C]-palmitate oxidation in a carnitine palmitoyl transferase-I (CPT-I)-dependent manner [51]. Therefore, ESP-55016 modulates both fatty acid and sterol synthesis and fatty acid oxidation through the ACC/malonyl-CoA/CPT-I axis.

#### 3.3.4. TEI-B00422

TEI-B00422, also called benzofuranyl alpha-pyrone, is a novel ACC inhibitor identified by screening for the inhibitory compounds of triglyceride (TG) synthesis from [14C] acetate in human hepatoma cell line HepG2 [79]. TEI-B00422 inhibits the incorporation of acetate into the triglyceride (TG) in rat primary hepatocytes, but not that of oleate. TEI-B00422 inhibits rat hepatic ACCs in a competitive manner with respect to acety-CoA with a K(i) at 3.3 µM. Due to the unique structure different from other known ACC inhibitors, TEI-B00422 may be utilized as a backbone in the design and development of novel therapeutic agents for metabolic disorders.

#### 3.3.5. MEDICA 16

MEDICA 16 (β,β′-tetramethyl hexadecanedioic acid) is a long-chain fatty acyl analog developed as a hypolipidemic and antiobesity-antidiabetogenic agent [38]. MEDICA 16 inhibits ACC activity and reduces AMPK activity in the liver, but not significantly in the skeletal muscle, which may be due to its poor penetration in muscle cells. In the JCR:LA-cp and Zucker rat, acute exposure of MEDICA 16 leads to significant inhibition of hepatic lipogenesis and decrease in plasma VLDL cholesterol and triglycerides [80,81], but chronic treatment prevents insulin resistance by inhibiting liver ACC activity [82,83]. In cell culture, MEDICA 16 decreases mitochondrial proton motive force, accompanied by an increase in cellular respiration [84].

#### 3.3.6. Chloroacetylated Biotin

Chloroacetylated biotin inhibit enzyme activity of ACCs and 3T3-L1 cell differentiation in a dose-dependent manner [85]. Chloroacetylated biotin also blocks the expression of PPARγ, STAT1, and STAT5A induced by adipogenesis. Moreover, chloroacetylated biotin can inhibit lipid accumulation, suggesting its potential as a herapeutic agent for anti-obesity therapy.

#### 3.3.7. S-2E

S-2 [(+/−)-4-[1-(4-tert-butylphenyl)-2-oxo-pyrrolidine-4-yl]methyloxybenzoic acid] is an anti- lipidemic agent with fatty acid and sterol biosynthesis inhibiting activity, lowering blood cholesterol and triglyceride levels [86]. The active form of S-2 is S-2E [(+)-(*S*)-*p*-[1-(p-tert-butylphenyl)-2-oxo-4-pyrrolidinyl] methoxybenzoic acid]-CoA, exerting non-competitive inhibition on ACCs at Ki = 69.2 µM [86]. S-2E-CoA is sufficient to inhibit the activity of HMG-CoA reductase and ACCs, when administered at 10 mg/kg orally in rabbits, suggesting that S-2E may be effective in the treatment of familial hypercholesterolemia [87,88].

#### 3.3.8. 4m-(S)

A high throughput screening of ACC1/2 pharmacological inhibitors identified a lead compound 3 (N-{(S)-3-[2-(4-isopropoxy-phenoxy)-thiazol-5-yl]-1-methyl-prop-2-ynyl}-acetamide) with moderate selectivity to ACC2 [89]. Its derivative 4m possesses dual ACC1/2 inhibitory activity in rats and humans at sub-micromolar levels [90]. Animal studies have shown that 4m induces fat oxidation and reduces plasma triglyceride levels, indicating its potential in the treatment of metabolic syndromes.

## 4. ACC Inhibitors in Cancer Therapy

Enhanced lipogenesis is a main feature of cancer cells, and the lipids newly synthesized in cells are implicated in cell migration, signal transduction, and intracellular trafficking [91,92,93,94,95,96,97]. As a rate-limiting enzyme of fatty acid synthesis, ACC1 has been found to be overexpressed in human cancers, such as breast, prostate, and liver carcinoma, even in ductal carcinoma *in situ* (DCIS) and lobular carcinoma *in situ* (LCIS) of breast and in PIN lesions of prostate [98,99,100,101,102,103]. In hepatocellular carcinoma, ACC1 is overexpressed with other two lipogenic enzymes, fatty acid synthase and ATP citrate lyase [104]. ACC1 alleles with variations in regulatory regions are found to affect ACC1 expression and associate with the breast cancer risk [105]. RNAi (RNA interference)-mediated ACC1 silencing leads to apoptosis of breast, colon and prostate cancer cells by reducing fatty acid synthesis [106,107]. These data suggest that ACC1 is universally upregulated in cancer cells to meet the enhanced needs for lipids, signaling molecules and energy consumption, thus being a promising target for cancer therapy [45,107,108].

It has been reported that the ACC inhibitor soraphen A blocks fatty acid synthesis and stimulate fatty acid oxidation in prostate cancer cells LNCaP and PC-3M at nanomolar levels, and thus exhausts phospholipid contents of cancer cells and suppresses cells proliferation, ultimately leading to cell death through apoptosis and autophagy pathways [78]. Our work showed that ACC inhibitor TOFA triggers apoptosis of lung (NCI-H460) and colon (HCT-8 and HCT-15) carcinoma cells through blocking fatty acid synthesis [76]. Furthermore, our studies also demonstrated that AKR1B10 mediates ACC1 stability and increase fatty acid synthesis; knockdown of AKR1B10 inhibits cancer cell growth and triggers apoptosis [55,56,57]. CP-640186 is a non-selective agent equally inhibiting both ACC1 and ACC2 in rat, mouse, monkey, and humans at an IC_50_ of 60 nM, and thus in animals, this compound decreases tissue malonyl-CoA levels, inhibits fatty acid biosynthesis and stimulates fatty acid oxidation, thus reducing body fat mass and body weight. These data support an opinion that isoform non-selective inhibitors might be adequate or even superior in metabolic syndrome treatment [37].

Malonyl-CoA generated by ACC2 at the mitochondrial membrane regulates fatty acid oxidation. Increased energy consumption and fatty acid oxidation represents a common feature of cancer cells, and thus inhibition of fatty acid oxidation might be a feasible approach for cancer therapy. Consistent with this concept, fatty acid oxidation inhibitors etomoxir and ranolazine inhibit leukemia cell proliferation [109], and trimetazidine induces cancer cell apoptosis [110]. Zhou *et al.* reported that malonyl-CoA accumulated by malonyl-CoA decarboxylase (MCD) inhibitors suppresses human breast cancer cell proliferation [111], and therefore reduction of fatty acid oxidation rates through the modulation of ACC2 activity appears to be a potential approach for the treatment of cancer. In addition, AMPK is a negative regulator of ACC activity [112] and activation of AMPK leads to ACC inhibition, decrease of malonyl-CoA level and increase of fatty acid and glucose oxidation, thus being a potential target for cancer management [113]. Nevertheless, although approaches targeted to ACCs have been explored and many small chemical inhibitors against ACC1 and/or ACC2 have been developed, their efficacy as potential drugs to cancer treatment remains to be further investigated. More isozyme-selective chemicals may need to be developed to improve anti-cancer activity. For instance, a combination of ACC1 inhibitors and ACC2 activators may be deserved to be explored for cancer treatment.

## 5. Conclusions

ACCs are important enzymes implicated in fatty acid synthesis and oxidation. Chemical genetic studies on ACCs have advanced the understanding of these enzymes in terms of the chemistry, biological and activity features. Most importantly, these studies have led to discovery and development of a series of natural and synthesized small chemical inhibitors of ACCs. Elevated ACC expression and lipogenesis in cancer cells confers its importance in cancer development and progression, as well as the potential as therapeutic targets of cancer. Preclinical studies in cell culture and experimental animals have set up a new milestone in developing novel agents for cancer treatment; and the serial studies on ACC inhibitors in metabolic syndromes may offer well documents for the translation of them into cancer therapy. However, due to the clear differences between the biological roles of ACC1 and ACC2 in fatty acid synthesis and oxidation, it is obvious that more selective inhibitors are needed to differentiate the clinical use. In addition, ACC1 mutations in breast cancer tissues lead to alterations of the binding and inhibitory activity of the inhibitors [108], and this needs to be taken into consideration in the development of novel ACC inhibitors as therapeutic agents of cancers.
